# 

**Published:** 1911-02-15

**Authors:** 


					﻿Dental
Register
NELVILLE S. HOFF, Editor,
Anri Arbor, Mich.
Vol. LXV. —FEBRUARY. 1911— No. 2
SAMUEL A. CROCKER & CO., Publishers
OHIO DENTAL AND SURGICAL DEPOT
35 W. FIFTH STREET, CINCINNATI, 0.
PRICE, ONE DOLLAR A YEAR
Entered at the Postoffice at Cincinnati, 0., as second-class mail matter.
				

